# Impact of temperature on the production of 1% polidocanol foam: comparison between the Tessari and double-syringe techniques in an experimental study

**DOI:** 10.1590/1677-5449.202500102

**Published:** 2025-08-01

**Authors:** Genaro Fahrnholz Buonsante, Marcos Arêas Marques, Bernardo Cunha Senra Barros, Verônica Cunha Assunção, Stênio Karlos Alvim Fiorelli, Rossano Kepler Alvim Fiorelli

**Affiliations:** 1 Universidade Federal do Estado do Rio de Janeiro – UNIRIO, Hospital Universitário Gaffrée e Guinle, Rio de Janeiro, RJ, Brasil.; 2 Universidade do Estado do Rio de Janeiro – UERJ, Hospital Universitário Pedro Ernesto – HUPE, Rio de Janeiro, RJ, Brasil.; 3 Hospital Municipal Salgado Filho – HMSF, Rio de Janeiro, RJ, Brasil.; 4 Academia Nacional de Medicina – ANM, Rio de Janeiro, RJ, Brasil.

**Keywords:** sclerotherapy, varicose veins, polidocanol, temperature, venous insufficiency, efficacy

## Abstract

**Background:**

Polidocanol (POL) foam is widely used in sclerotherapy for the treatment of lower limb varicose veins and its properties are influenced by multiple variables, including preparation methods and room temperature.

**Objectives:**

To compare the influence of temperature on the half-life and bubble diameter of 1% polidocanol foam using the Tessari and double syringe techniques.

**Methods:**

The study employed 1% polidocanol foam prepared at room temperature and cooled to 4 °C, using two techniques: the Tessari technique and the double syringe technique. The foam half-life was recorded, defined as the time in seconds taken for half of the liquid volume to drain. Bubble diameter was analyzed with microscopy. Differences between groups were considered significant at p ≤ 0.05.

**Results:**

Cooling significantly extended the half-life of the foam, especially when the double syringe technique was employed. The Tessari technique produced smaller bubbles under both temperature conditions.

**Conclusions:**

Cooling the mixture of 1% polidocanol and room air increased half-life, irrespective of the preparation technique employed. Temperature had no effect on bubble diameter.

## INTRODUCTION

Foam sclerotherapy(FS) is a widely established technique for treatment of lower limb (LL) varicose veins and vascular malformations.^1^ The FS offers the advantage of increased viscosity when compared to liquid, enabling better displacement of the intravascular content, increasing the surface area in contact with endothelium and reducing dilution and deactivation of the sclerosing agent by plasma.^2^ The technique involves intravenous infusion of a sclerosing agent, administered under direct view or guided by ultrasonography, and has been described with several different sclerosants and preparation methods. Polidocanol (POL) is the most widely used sclerosing agent in Brazil, offering the advantages of low cost and applicability to office procedures.^3-5^

Polidocanol is an amphiphilic surfactant molecule, comprising a hydrophilic polar component and an apolar hydrophobic component, with similar properties to phospholipid membranes. Its action reduces surface tension and, eventually, dissolves the endothelial cell membrane, provoking inflammation and fibrosis of the vessel. Foam can be defined as of adequate quality if it has a half-life of approximately 2 minutes and microbubbles with a diameter less than 250 µm.^6^

Polidocanol foam can be made by physicians using techniques such as the Tessari technique (TT) and the double syringe technique (DST).^7,8^ Standardized commercial preparations and automated preparation devices are also available on the market, such as Varithena^®^ (Boston Scientific, Marlborough, Massachusetts, United States) and Varixio^®^ (VB Devices, Barcelona, Spain), for example.^9,10^

The main criticisms of sclerotherapy performed using physician prepared foam relate to the lack of standardization and the large number of variables that can impact its quality. The most important of these are the pressure applied to the syringe plunger, the velocity of mixture preparation, different concentrations of sclerosing agent, the proportion of liquid to gas, the type of gas used, the preparation method (TT or DST), the quality of the materials employed, and local altitude and temperature.^2,11^

The pressure and velocity of preparation impact the rate of foam flow, which is determined by its viscosity. In turn, viscosity is affected by density (mass per unit of volume) and by shear forces (related to the velocity gradient within the diameter of the conduit). A fluid with viscosity that remains constant, irrespective of shear forces is classified as a Newtonian fluid. The viscosity of POL foam reduces as shear forces increase and so it is classified as a non-Newtonian fluid.^12-14^

Some of these variables, such as the POL concentration and the type of gas employed depend on factors related to the patient and may be adjusted depending on the location and size of the vessel being treated.^1^ The quality of the sclerosing agent, environmental variables, and factors related to the physician, such as altitude and the pressure applied during preparation, are difficult to standardize. In contrast, the preparation technique, the temperature, and use of specific materials are easily controlled and are reproducible.

It is worth mentioning that very often changing these variables does not add significant extra cost to the process, which is a relevant point, considering that foam sclerotherapy is widely used to treat LL varicose veins on the Brazilian Unified Health System (SUS - Sistema Único de Saúde).^15^

Studies suggest that use of nonsilicone syringes, employing the DST, and reducing the temperature all increase the stability of DF, which is assessed in terms of its half-life. Although there are studies that have compared these variables individually, suggesting that the DST and use of nonsilicone syringes increase the half-life of the foam, no studies were found that have investigated the influence of temperature in a scenario in which all of these characteristics were combined.^11,16,17^ Assessing these factors in conjunction would make it possible to assess the impact on the quality of physician prepared foam of low-cost variables that are easy to standardize.

The objective of this study is to analyze the quality of 1% POL foam prepared at room temperature and with cooling, using the TT and the DST, employing nonsilicone syringes.

## METHODS

An *in vitro* experimental laboratory study was conducted using the following materials: POL 1% (Victalab^®^ Farmácia de Manipulação Ltda., São Paulo, Brazil); 3 and 5 mL syringes with Luer Lock (SR Productos Para la Salud^®^, Pedro Juan Caballero, Paraguay); a three-way connector with Luer Lock (Poly Medcure Ltda., Haryana, India); a two-way connector (Baxter^®^ International Inc., Illinois, United States); a 25 G infusion port (Medix Brasil^®^, Paraná, Brazil); an Exbom digital internal and external thermo-hygrometer (Exbom^®^, São Paulo, Brazil); an Olympus CX 41 microscope (Olympus Corporation^®^, Tokyo, Japan); and an Electrolux EM120 refrigerator (Electrolux^®^, Paraná, Brazil).

### Preparation

**Figure 1:**
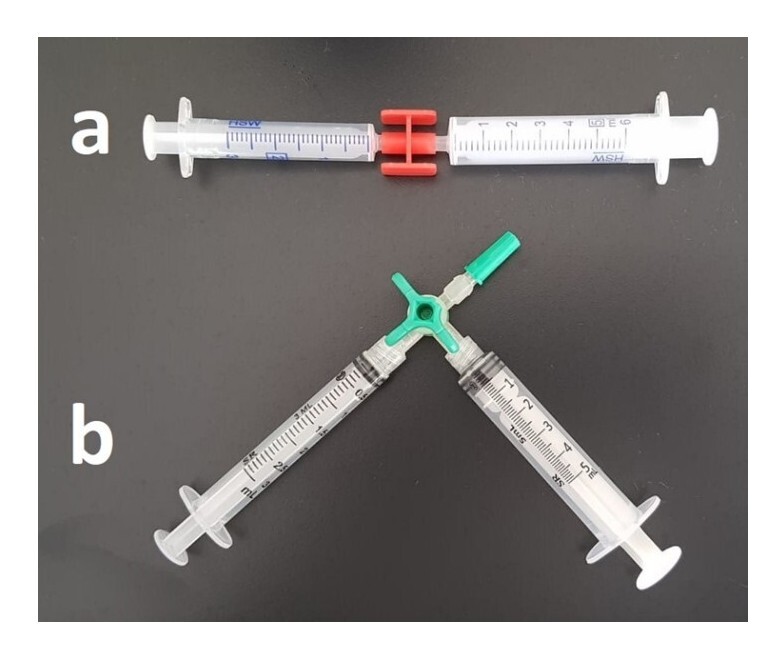
(a) Arrangement of syringes for the double syringe technique; (b) Arrangement of syringes for the Tessari technique.

The 1% POL foam was prepared at room temperature and with cooling using TT and DST, with nonsilicone syringes. For the TT, 3 and 5 mL syringes were connected at a 90º angle using a three-way connector (Figure 1a). For the DST, 3 and 5 mL syringes were connected at a 180 º angle using a two-way connector (Figure 1b).

All samples were prepared at a proportion of 1:4 with room air, i.e. 1 mL of POL 1% and 4 mL of air. The syringes were connected and shaken, executing 20 complete movements of the plungers and then measurements were taken immediately after preparation. Groups were formed without blinding and all experiments were performed by the same operator.

### Temperature control

The room temperature samples were prepared at 25 °C. The cooled samples were prepared after cooling the syringes, already filled with POL 1% and room air, to a temperature of 4±1 °C in a refrigerator. Temperature was not measured again after preparation of the foam, so that the outcome data could be measured as close to immediately as possible. The time taken to cool the samples was defined empirically as 15 minutes in pilot tests.

### Measurement of half-life

**Figure 2:**
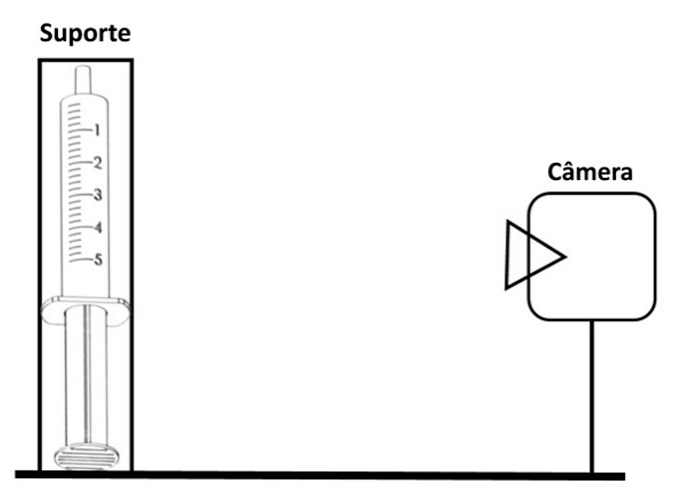
Diagram illustrating positioning of materials to assess half-life.

The foam half-life was measured in seconds for all experimental groups. Half-life was defined as the time taken for half of the volume of the liquid to drain. Timing began immediately after completion of the preparation, with the syringe positioned vertically in a support, and was video recorded (Figure 2). Each experiment was repeated 10 times. The number of repetitions was determined by using the largest number of repetitions reported in similar experiments.^8-10,18,19^ Times are reported as the means of measurements in seconds with their standard deviations.

### Measurement of bubble diameter

These measurements were performed immediately after each preparation was complete. A 0.5 mL volume of foam was transferred to a slide using a 25 G infusion port and covered with a coverslip. Three different areas of the sample were photographed with a microscope fitted with a 2.5x eyepiece and using a 10x objective lens. Five samples were prepared for each experimental group.

**Figure 3:**
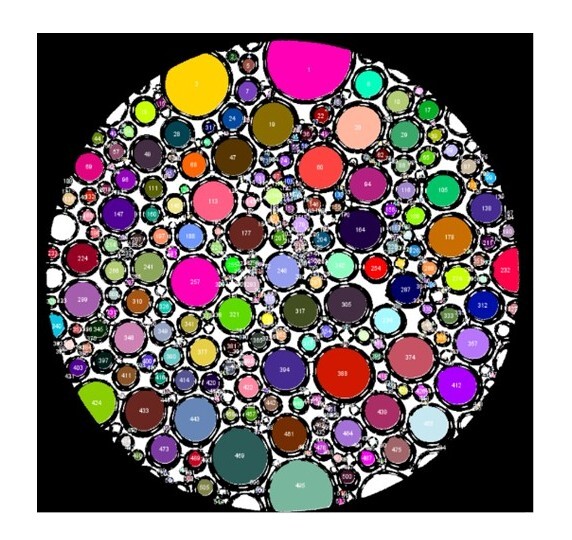
Image of bubbles after processing by analysis software.

Bubble diameters are reported as means, in microns, and their standard deviations. For image processing, an image of a calibration slide was captured to define the scale and Fiji-ImageJ **^®^** was used to analyze the images (Figure 3).^20^

### Statistical

The Shapiro-Wilk test was used to verify the normality of each dataset, adopting a significance level (p) of ≤ 0.05. For data considered normal, the Levene test was used to verify the homogeneity of variance in the groups.

If both conditions were met, the ANOVA test, with Tukey *post hoc* analysis was used to identify which pairs of groups were significantly different. If the Shapiro-Wilk test or the Levene test indicated that the premises of distribution normality or variance homogeneity were violated, the Kruskal-Wallis test was used instead, with the Dunn test *post hoc* analysis applied to identify significantly different pairs. Results were interpreted on the basis of their p values, adopting a significance level of p ≤ 0.05.

## RESULTS

### Half-life

Table 1 shows the mean half-lives and the statistical analysis results, demonstrating statistically significant differences between the groups analyzed (p < 0.01).

**Table 1:** Half-lives and statistical analysis.

**Group (n)**	**Mean (s)**	**SD (s)**
Room temperature TT (10)	90	8.57
Room temperature DST (10)	148	37.71
Cooled TT (10)	132	10.71
Cooled DST (10)	207	34.63
***Post hoc* analysis to compare groups**		**P value**
Room temperature vs. DST at room temperature		< 0.01
Room temperature TT vs. cooled TT		< 0.01
Room temperature TT vs. cooled DST		< 0.01
Room temperature DST vs. cooled TT		0.63
Room temperature SD vs. cooled DST		< 0.05
Cooled TT vs. cooled DST		< 0.05

TT = Tessari technique; DST = double syringe technique; SD = standard deviation; s = seconds.

Table 2 shows mean bubble diameters, in microns, and the results of the statistical analysis. Statistically significant differences were observed between the different preparation techniques, but temperature had no effect on bubble diameter.

**Table 2:** Bubble diameter and statistical analysis.

**Group (n)**	**Mean (μm)**	**Standard deviation (μm)**	**95%CI**
Room temperature TT (1,236)	53.50	11.95	±6.62
Room temperature DST (2,884)	86.54	19.92	±11.03
Cooled TT (2,455)	52.81	21.82	±12.09
Cooled DST (1,668)	74.20	21.11	±11.69
***Post hoc* analysis to compare groups**			**Value p**
Room temperature TT vs. room temperature DST			< 0.01
Room temperature TT vs. cooled TT			0.74
Room temperature TT vs. cooled DST			0.01
Room temperature DST vs. cooled TT			< 0.01
Room temperature DST vs. cooled DST			0.22
Cooled TT vs. cooled DST			< 0.01

TT = Tessari technique; DST = double syringe technique; 95%CI = 95% confidence interval.

**Figure 4:**
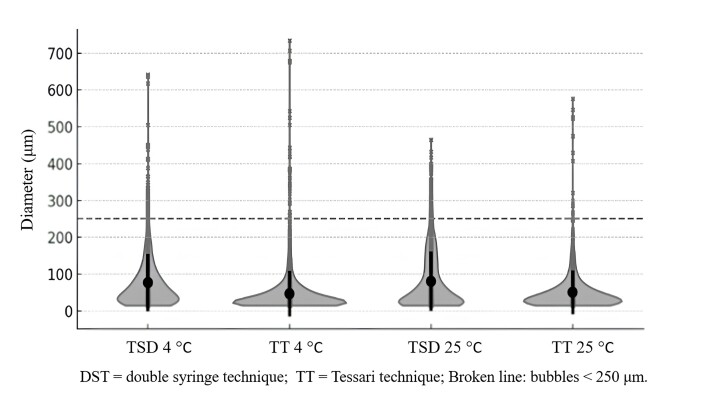
Distribution of bubble sizes.

Figure 4 illustrates the proportions of bubbles with diameters less than 250 µm and the distribution of bubble diameters in foam prepared with the different techniques and under different conditions. The TT at room temperature had the smallest proportion (1.21%) and the DST at the same temperature had the largest proportion (4.26%).

## DISCUSSION

Preparation of foam by the physician is subject to considerable technical variability. Several factors influence its quality and, while there are experimental studies demonstrating how each variable impacts the foam, some are not easily reproducible or controllable outside the laboratory.^11^

Temperature is one of the factors that influence preparation of foam and can be controlled without highly expensive or complex equipment. There is literature demonstrating a correlation between low temperatures, varying from 4 to 10 °C, and an increase in foam half-life, resulting in greater stability because of the increased viscosity.^11,16,21^ This is why it is important to mention the influence not only of the temperature of the POL itself, but also of all of the materials involved in foam preparation.^17^

Commercial preparations have been developed to attenuate the effect of these variables. Varithena **^®^** POL microfoam (PMF) and the Varixio **^®^** preparation device are currently available. The former uses a preparation of 1% POL mixed with a predefined mixture of O_2_ and CO_2_. Carugo et al.^10^ compared the half-life of PMF with foams prepared with TT and DST at different concentrations and varying gas mixtures, observing a half-life of approximately 120 seconds for PMF, around 160 seconds for DST, and 90 seconds for TT, all produced at room temperature.

Varixio **^®^** employs a magnetic mixer and a capsule to produce the mixture and can be used with POL at different concentrations to mix with air or O_2_/CO_2_. The half-life for 1% POL, as used in the present study, was approximately 144 seconds and the mean bubble diameter was 86±14 µm.^9^

With regard to half-life, mean half-life at room temperature was 90 seconds for the TT preparation. This value is compatible with other studies, which report mean half-lives ranging from 95 to 145 seconds, using the same parameters for dilution, temperature, and liquid-to-air ratio.^8,9,16,18^

In addition to the variables already mentioned, the difference may also be attributable to variations in the velocity of preparation between different physicians. Bai et al.^21,22^ analyzed the influence on foam of preparation velocity using an automated mixing device. Velocities from 100 to 350 mm/s had a significant impact, with the maximum half-life achieved at 275 mm/s, reducing at higher velocities. This parameter is difficult to standardize across different professionals and, although the samples were all prepared by the same professional, this study is subject to such variations, which emphasizes the need for methods of preparation optimization that are easy to standardize outside of the laboratory setting.

Methods of foam optimization that are easy to standardize are important, considering that foam is now an established and cost-effective technique for treatment of LL varicose veins and venous ulcers.^23,24^

Temperature can be controlled without the need for expensive equipment. The time taken for cooling is an important variable in the process and in the present study the time observed for the apparatus to cool was 15 minutes. After cooling, the foam preparation itself causes an increase in temperature that is dependent on the initial temperature, with experiments showing that it is necessary to cool the entire system to a temperature of 4 ^o^C to achieve a post-preparation temperature of 10 ^o^C.^17^

Valenzuela et al.^16^ conducted an experimental study and reported a half-life of approximately 150 seconds, for POL at both a concentration of 0.5% and a concentration of 1.5%, at a temperature of 10 °C. It should be mentioned that they used a 5 µm filter in the circuit, which could have contributed to increased half-life because of the increased shear forces.^22^

When we analyzed the data obtained with the DST using nonsilicone syringes at room temperature, the half-life recorded was 148 seconds. Shi et al.^8^ reported values of 142.27±2.98 seconds, which were statistically longer than times obtained using the TT in the same study.

It is important to mention that those authors used siliconized syringes and observed similar half-lives. It would therefore be interesting to conduct a detailed study of the true impact of using siliconized syringes and nonsilicone syringes, considering that there are few publications that have directly compared these two approaches.^11^

The longest mean half-life of 1% POL foam in this study was achieved using the DST with cooled nonsilicone syringes, with a half-life of 207±34 seconds. This is comparable to the half-lives of commercial preparations, such as a preparation of POL 1% + room air, which had a half-life of 144±54 seconds.^9,10^

When bubble diameter was analyzed, temperature did not cause significant differences, either with the TT or with the DST. However, there were statistically significant differences in mean bubble diameter, with the TT foam having a smaller mean diameter and a lower proportion of microbubbles (< 250 µm) compared to the DST foam. These findings diverge from results observed by Shi et al.,^8^ who reported that the TT resulted in a smaller mean bubble diameter than the DST.^22^

Despite the divergence, this study demonstrates, as other authors have reported, that both techniques for physician prepared foam are capable of consistently producing microbubbles comparable to commercial preparations.^2,9,10^

In terms of clinical applicability, this study is relevant, since foam plays an important role in treatment of LL varicose veins^23,24^ and more stable foam offers greater flexibility in terms of the time available for administration. Nevertheless, this study has the limitation of an *in vitro* analysis, which makes it impossible to correlate the greater foam stability with clinical benefit. Another limiting factor is the number of technical variations that exist, such as an angled three-way valve or inclusion of valves and filters between syringes, which impairs comparisons between techniques. It is also known that POL concentration, the proportion of liquid to room air, and the variation of foam temperature in relation to body temperature negatively affect DF stability.^25,26^ One avenue of interest for future studies would be to compare the influence of temperature at concentrations lower than 1%.

## CONCLUSIONS

Cooling both the 1% POL and the room air led to an increase in foam half-life, irrespective of the preparation technique used. Temperature had no influence on bubble diameter.
