# Supplementary material for: Impact of temperature on the production of 1% polidocanol foam: comparison between the Tessari and double-syringe techniques in an experimental study
Source: J Vasc Bras. 2025 Aug 1;24:e20250010. doi: 10.1590/1677-5449.202500102 (PMC12419747; doi:10.1590/1677-5449.202500102)
Supplement: Translation

# Translation: Impacto da temperatura na produção de espuma densa de polidocanol a 1%: comparação entre as técnicas de Tessari e seringa dupla em estudo experimental

**DOI:** 10.1590/1677-5449.202500101

**Authors:** Genaro Fahrnholz Buonsante, Marcos Arêas Marques, Bernardo Cunha Senra Barros, Verônica Cunha Assunção, Stênio Karlos Alvim Fiorelli, Rossano Kepler Alvim Fiorelli

**Affiliations:** 1 Universidade Federal do Estado do Rio de Janeiro – UNIRIO, Hospital Universitário Gaffrée e Guinle, Rio de Janeiro, RJ, Brasil.; 2 Universidade do Estado do Rio de Janeiro – UERJ, Hospital Universitário Pedro Ernesto – HUPE, Rio de Janeiro, RJ, Brasil.; 3 Hospital Municipal Salgado Filho – HMSF, Rio de Janeiro, RJ, Brasil.; 4 Academia Nacional de Medicina – ANM, Rio de Janeiro, RJ, Brasil.

**Keywords:** escleroterapia, varizes, polidocanol, temperatura, insuficiência venosa, eficácia

## Abstract

**Contexto:**

A espuma densa de polidocanol é amplamente utilizada na escleroterapia para o tratamento de varizes de membros inferiores, sendo influenciada por diversas variáveis nos métodos de preparo e pela temperatura ambiente.

**Objetivos:**

Comparar a influência da temperatura na meia-vida e no diâmetro das bolhas na espuma densa de polidocanol a 1% utilizando as técnicas de Tessari e da seringa dupla.

**Métodos:**

O estudo utilizou espuma densa de polidocanol a 1%, preparada em temperatura ambiente e resfriada a 4 °C, por meio de duas técnicas: técnica de Tessari e técnica da seringa dupla. A meia-vida em segundos da espuma densa foi avaliada registrando-se o tempo necessário para a drenagem de metade do volume líquido, e o diâmetro das bolhas foi analisado por microscopia. As diferenças entre os grupos foram consideradas significativas quando p ≤ 0,05.

**Resultados:**

O resfriamento aumentou significativamente a meia-vida da espuma densa, especialmente quando a técnica da seringa dupla foi utilizada. A técnica de Tessari produziu bolhas menores em ambas as condições de temperatura.

**Conclusões:**

O resfriamento do conjunto de polidocanol a 1% + ar ambiente gerou aumento da meia-vida, independentemente da técnica de preparo utilizada. Não houve influência da temperatura no diâmetro das bolhas.

## INTRODUÇÃO

A escleroterapia com espuma densa (ED) é uma técnica amplamente estabelecida para o tratamento de varizes dos membros inferiores (MMII) e de malformações vasculares^1^ . A ED apresenta como vantagem o aumento da viscosidade quando comparada à forma líquida, permitindo um maior deslocamento do conteúdo intravascular, ampliando a superfície de contato com o endotélio e reduzindo a diluição e a desativação do agente esclerosante pelo plasma^2^ . Essa técnica envolve a infusão intravenosa de um esclerosante, realizada sob visão direta ou guiada por ultrassonografia, sendo descrita com o uso de diferentes esclerosantes e métodos de preparação. O polidocanol (POL) é o esclerosante mais utilizado no Brasil, apresentando como vantagens o baixo custo e a viabilidade para procedimentos ambulatoriais^3-5^ .

O POL é uma molécula surfactante anfifílica, composta por um componente polar hidrofílico e um componente apolar hidrofóbico, apresentando propriedades semelhantes às da membrana fosfolipídica. Sua ação consiste em reduzir a tensão superficial e, em última instância, solubilizar a membrana celular do endotélio, levando à inflamação e fibrose do vaso. Uma espuma de qualidade adequada pode ser definida por uma meia-vida de aproximadamente 2 minutos e microbolhas com diâmetro inferior a 250 µm^6^ .

A ED de POL pode ser obtida por meio de técnicas de preparo realizadas pelo médico, como a técnica de Tessari (TT) e a técnica da seringa dupla (TSD)^7,8^ . Também estão disponíveis no mercado preparações comerciais padronizadas e dispositivos automatizados para o preparo, como o Varithena^®^ (Boston Scientific, Marlborough, Massachusetts, EUA) e o Varixio^®^ (VB Devices, Barcelona, Espanha)^9,10^ .

As principais críticas à escleroterapia com ED preparada manualmente pelos médicos referem-se à falta de padronização e ao grande número de variáveis que podem influenciar sua qualidade. Entre essas, destacam-se a pressão aplicada no êmbolo da seringa, a velocidade de preparo da mistura, as diferentes concentrações do esclerosante, a proporção líquido-gás, o tipo de gás utilizado, as formas de preparo (TT ou TSD), a qualidade do material empregado, bem como a altitude e a temperatura locais^2,11^ .

A pressão e a velocidade de preparo influenciam o fluxo da ED, que é determinado por sua viscosidade. A viscosidade, por sua vez, é afetada pela densidade (massa por unidade de volume) e pelas forças de cisalhamento (relacionadas ao gradiente de velocidade dentro do diâmetro do conduto). Um fluido cuja viscosidade permanece constante, independentemente das forças de cisalhamento, é classificado como um fluido newtoniano. A ED de POL apresenta redução de viscosidade com o aumento das forças de cisalhamento, sendo classificada como um fluido não newtoniano^12-14^ .

Algumas dessas variáveis, como a concentração de POL e o tipo de gás utilizado, dependem de fatores relacionados ao paciente e podem variar conforme o local e o tamanho do vaso a ser tratado^1^ . A qualidade do esclerosante, as variáveis ambientais e os fatores relacionados ao ato médico, como a altitude e a pressão aplicada durante a preparação, são aspectos de difícil padronização. Em contrapartida, a técnica de preparo, a temperatura e o uso de materiais específicos são fatores facilmente controláveis e reprodutíveis.

Vale mencionar que a modificação dessas variáveis, muitas vezes, não acarreta aumento significativo nos custos do processo, o que é um ponto relevante, considerando que a escleroterapia com ED é amplamente utilizada no tratamento de varizes de MMII no contexto do Sistema Único de Saúde (SUS) brasileiro^15^ .

Alguns estudos sugerem que o uso de seringas não siliconadas, o uso da TSD e a redução da temperatura aumentam a estabilidade da ED, avaliada através de sua meia-vida. Embora existam estudos que compararam essas variáveis individualmente, sugerindo que a TSD e o uso de seringas não siliconadas aumentam a meia-vida da ED, não foram encontrados trabalhos que investigassem a influência da temperatura em um cenário onde todas essas características fossem combinadas^11,16,17^ . Ao se considerar a associação desses fatores, torna-se possível avaliar o impacto de variáveis de baixo custo e fácil padronização na qualidade da ED produzida pelo médico.

O objetivo deste estudo é analisar a qualidade da ED de POL a 1% preparada em temperatura ambiente e sob resfriamento, utilizando a TT e TSD, com seringas não siliconadas.

## MÉTODOS

Foi realizado um estudo experimental laboratorial *in vitro* utilizando os seguintes materiais: POL a 1% (Victalab^®^ Farmácia de Manipulação Ltda., São Paulo, Brasil); seringas de 3 e 5 mL com Luer Lock (SR Productos Para la Salud^®^, Pedro Juan Caballero, Paraguai); conector de três vias com Luer Lock (Poly Medcure Ltda., Haryana, Índia); conector de duas vias (Baxter^®^ International Inc., Illinois, Estados Unidos); escalpe para infusão 25 G (Medix Brasil^®^, Paraná, Brasil); termo-higrômetro digital interno e externo Exbom (Exbom^®^, São Paulo, Brasil); microscópio Olympus CX 41 (Olympus Corporation^®^, Tóquio, Japão); e refrigerador Eletrolux EM120 (Eletrolux^®^, Paraná, Brasil).

### Preparação

A ED de POL a 1% foi preparada em temperatura ambiente e resfriada utilizando a TT e a TSD, com seringas não siliconadas. Na TT, seringas de 3 e 5 mL foram conectadas por meio de um conector de três vias em um ângulo de 90º (Figura 1a). Na TSD, seringas de 3 e 5 mL foram conectadas por meio de um conector de duas vias em um ângulo de 180º (Figura 1b).

**Figura 1:** (a) Disposição das seringas para realização da técnica da seringa dupla; (b) Disposição das seringas para realização da técnica de Tessari.

Todas as amostras foram preparadas utilizando a proporção de 1+4 com ar ambiente, sendo 1 mL de POL a 1% e 4 mL de ar. As seringas foram conectadas e agitadas com 20 movimentos completos do êmbolo, e as aferições foram realizadas imediatamente após a preparação. A escolha dos grupos foi feita de forma aberta e o experimento realizado pelo mesmo operador.

### Controle de temperatura

As amostras em temperatura ambiente foram preparadas a 25 °C. As amostras resfriadas foram preparadas resfriando a seringa, já contendo o POL a 1% e ar ambiente, a uma temperatura de 4±1 °C, utilizando um refrigerador. A temperatura após a preparação da espuma não foi aferida para que a medição dos dados ocorresse o mais imediatamente possível. O tempo necessário para o resfriamento da amostra foi definido empiricamente através de testes-piloto, sendo estabelecido em 15 minutos.

### Medição da meia-vida

Para todos os grupos, foi registrada a meia-vida da preparação em segundos, definida como o tempo necessário para a drenagem de metade do volume de líquido utilizado. A cronometragem foi iniciada imediatamente após o término da preparação, com a seringa posicionada verticalmente em um suporte para registro em vídeo (Figura 2). Foram realizadas 10 repetições. O número de repetições foi determinado utilizando como base o maior número descrito em experimentos semelhantes^8-10,18,19^ . Os dados de tempo são apresentados através das médias das medidas em segundos e seus desvios-padrão.

**Figura 2:** Diagrama do posicionamento dos materiais para avaliação da meia-vida.

### Medição do diâmetro das bolhas

A medição foi realizada imediatamente após o término da preparação. Um volume de 0,5 mL de ED foi transferido para uma lâmina utilizando um escalpe de infusão 25 G e coberto com uma lamínula. Três áreas distintas da amostra foram registradas por meio de uma câmera acoplada a um microscópio equipado com ocular de 2,5x e objetiva de 10x. Para cada grupo, foram preparadas cinco amostras.

Os diâmetros das bolhas são apresentados pela média das medidas em micrômetros e seus desvios-padrão. Para o processamento das imagens, foi realizada uma captura de imagem de uma lâmina de calibração para definição da escala, e o *software* de análise de imagens Fiji-ImageJ **^®^** foi utilizado (Figura 3)^20^ .

**Figura 3:** Imagem das bolhas após processamento no *software* de análise.

### Estatística

O teste de Shapiro-Wilk foi aplicado a cada conjunto de dados para avaliar sua normalidade, adotando um nível de significância (p) ≤ 0,05. Para os dados considerados normais, o teste de Levene foi utilizado para verificar a homogeneidade das variâncias entre os grupos.

Se ambas as condições fossem atendidas, realizava-se o teste ANOVA, com análise *post hoc* de Tukey para identificar quais pares de grupos apresentavam diferenças significativas. Caso o teste de Shapiro-Wilk ou o teste de Levene indicassem violação das premissas de normalidade ou homogeneidade das variâncias, o teste de Kruskal-Wallis era empregado como alternativa, com análise *post hoc* pelo teste de Dunn para identificar os pares com diferenças significativas. Os resultados foram interpretados com base nos valores de p obtidos, considerando um nível de significância ≤ 0,05.

## RESULTADOS

### Tempo de meia-vida

A média do tempo de meia-vida e a análise estatística são apresentadas na Tabela 1, evidenciando diferença estatisticamente significativa entre os grupos avaliados (p < 0,01).

**Tabela 1:** Tempo de meia-vida e análise estatística.

**Grupo (n)**	**Média (s)**	**DP (s)**
Temperatura ambiente TT (10)	90	8,57
Temperatura ambiente TSD (10)	148	37,71
Resfriado TT (10)	132	10,71
Resfriado TSD (10)	207	34,63
**Análise *post hoc* para comparação entre grupos**		**Valor de p**
Temperatura ambiente TT vs. temperatura Ambiente TSD		< 0,01
Temperatura ambiente TT vs. resfriado TT		< 0,01
Temperatura ambiente TT vs. resfriado TSD		< 0,01
Temperatura ambiente TSD vs. resfriado TT		0,63
Temperatura ambiente SD vs. resfriado TSD		< 0,05
Resfriado TT vs. resfriado TSD		< 0,05

TT = técnica de Tessari; TDS = técnica de seringa dupla; DP = desvio-padrão; s = segundos.

O diâmetro médio das bolhas, em micrômetros, e a análise estatística são apresentados na Tabela 2. Foram observadas diferenças estatisticamente significativas entre as técnicas de preparação, sem influência da temperatura no diâmetro das bolhas.

**Tabela 2:** Diâmetro das bolhas e análise estatística.

**Grupo (n)**	**Média (μm)**	**Desvio-padrão (μm)**	**IC95%**
Temperatura ambiente TT (1.236)	53,50	11,95	±6,62
Temperatura ambiente TSD (2.884)	86,54	19,92	±11,03
Resfriado TT (2.455)	52,81	21,82	±12,09
Resfriado TSD (1.668)	74,20	21,11	±11,69
**Análise *post hoc* para comparação entre grupos**			**Valor p**
Temperatura ambiente TT vs. temperatura Ambiente TSD			< 0,01
Temperatura ambiente TT vs. resfriado TT			0,74
Temperatura ambiente TT vs. resfriado TSD			0,01
Temperatura ambiente TSD vs. resfriado TT			< 0,01
Temperatura Ambiente TSD vs. resfriado TSD			0,22
Resfriado TT vs. resfriado TSD			< 0,01

TT = técnica de Tessari; TDS = técnica de seringa dupla; IC95% = intervalo de confiança de 95%.

A Figura 4 mostra a proporção de bolhas menores que 250 µm e a distribuição de bolhas nas diferentes técnicas e condições, com a TT em temperatura ambiente apresentando a menor proporção (1,21%) e a TSD na mesma condição apresentando a maior (4,26%).

**Figura 4:** Distribuição dos tamanhos das bolhas.

## DISCUSSÃO

A preparação da ED, realizada pelo médico, está sujeita a uma grande variabilidade técnica. Diversos fatores influenciam a sua qualidade e, embora existam estudos experimentais que detalhem como cada variável interfere nela, algumas não são facilmente reprodutíveis ou controláveis fora do ambiente laboratorial^11^ .

A temperatura é um dos fatores que influenciam a preparação da ED e pode ser controlada sem a necessidade de equipamentos de alto custo ou complexidade. A literatura demonstra uma correlação entre temperaturas baixas, variando de 4 a 10 °C, e um aumento na meia-vida da ED, resultando em maior estabilidade devido ao aumento na viscosidade^11,16,21^ . Por isso, é importante mencionar a influência não apenas da temperatura do POL em si, mas também de todos os materiais envolvidos na preparação^17^ .

Como forma de atenuar essas variáveis, foram desenvolvidas preparações comerciais, estando atualmente disponíveis a microespuma (MEP) de POL (Varithena **^®^** ) e o Varixio **^®^** . A primeira utiliza uma preparação de POL a 1% com uma mistura predefinida de O_2_ e CO_2_. Carugo et al.^10^ compararam a meia-vida da MEP com as TT e TSD em diferentes concentrações e misturas de gases, obtendo uma meia-vida de aproximadamente 120 segundos para a MEP, cerca de 160 segundos para a TSD e 90 segundos para a TT, todas em temperatura ambiente.

O Varixio **^®^** utiliza um misturador magnético e uma cápsula para gerar a mistura, permitindo o uso de POL em diferentes concentrações com ar ou O_2_/CO_2_. Para o POL a 1%, conforme utilizado neste estudo, a meia-vida foi de aproximadamente 144 segundos, com um diâmetro médio de bolhas de 86±14 µm^9^ .

Em relação à meia-vida, foi observado, em temperatura ambiente, uma média de 90 segundos para a preparação utilizando a TT. O valor é compatível com outros estudos, que relatam uma meia-vida média de 95 a 145 segundos utilizando os mesmos padrões de diluição, temperatura e proporção líquido + ar^8,9,16,18^ .

Além das variáveis já citadas, essa diferença também pode ser atribuída à variação na velocidade de preparo da ED entre os médicos. Bai et al.^21,22^ avaliaram a influência da velocidade de preparação da ED utilizando um dispositivo de mistura automatizado. Velocidades entre 100 e 350 mm/s tiveram impacto significativo, com a meia-vida máxima alcançada a 275 mm/s, reduzindo em velocidades superiores. Esse parâmetro é de difícil padronização entre profissionais e, embora o preparo das amostras tenha sido realizado sempre pelo mesmo profissional, este trabalho está sujeito a variações, o que reforça a necessidade de formas de otimização do preparo que sejam de fácil padronização fora do ambiente laboratorial.

Formas de otimização da espuma que sejam de fácil padronização são importantes, considerando que a ED é uma técnica consagrada e custo-efetiva para o tratamento de varizes e úlceras venosas de MMII^23,24^ .

O controle da temperatura pode ser realizado sem a necessidade de equipamentos de alto custo. O tempo necessário para o resfriamento é uma variável importante no processo, sendo observado, neste trabalho, um tempo de 15 minutos para o resfriamento do conjunto. Após o resfriamento, a própria preparação da espuma gera um aumento de temperatura dependente da temperatura inicial, com experimentos mostrando que é necessário resfriar todo o sistema a uma temperatura de 4 °C para atingir uma temperatura pós-preparo de 10 °C^17^ .

Valenzuela et al.^16^ , em um estudo experimental, relataram uma meia-vida de aproximadamente 150 segundos, tanto para concentrações de POL a 0,5% quanto a 1,5%, a uma temperatura de 10 °C. Ressalta-se que esses autores utilizaram um filtro de 5 µm no circuito, o que pode ter contribuído para o aumento da meia-vida devido ao incremento das forças de cisalhamento^22^ .

Ao analisarmos os dados obtidos com a TSD utilizando seringas não siliconadas em temperatura ambiente, foi registrada uma meia-vida de 148 segundos. Shi et al.^8^ relataram valores de 142,27±2,98 segundos, estatisticamente maiores do que os obtidos no mesmo estudo para a TT.

É importante destacar que os autores utilizaram seringas siliconadas, obtendo valores semelhantes de meia-vida. Portanto, seria interessante a realização de um estudo aprofundado sobre o impacto real do uso de seringas siliconadas e não siliconadas, considerando que existem poucas referências que comparam diretamente essas duas abordagens^11^ .

A maior meia-vida média da ED de POL a 1% neste estudo foi alcançada utilizando a TSD com seringa não siliconada e resfriada, com uma T1/2 de 207±34 segundos. Esse valor é comparável à meia-vida de preparações comerciais, como a do POL a 1% + ar ambiente, que apresentou uma meia-vida de 144±54 segundos^9,10^ .

Quando analisado o diâmetro das bolhas, a temperatura não apresentou diferença significativa, tanto na TT quanto na TSD. No entanto, houve diferença estatisticamente significativa no diâmetro médio das bolhas, com a TT apresentando uma média menor e uma menor proporção de microbolhas (< 250 µm) em comparação à TSD. Esses dados divergem dos resultados encontrados por Shi et al.^8^ , que relataram que a TT apresenta um diâmetro médio de bolhas menor que a TSD^22^ .

Apesar da divergência, este estudo demonstra, assim como outros autores já relataram, que ambas as técnicas de ED preparadas pelo médico são capazes de produzir uma ED com microbolhas de forma consistente e comparável às preparações comerciais^2,9,10^ .

Em relação à aplicabilidade clínica, este estudo é relevante, uma vez que a ED tem papel importante no tratamento das varizes de MMII^23,24^ , e uma ED mais estável proporciona maior flexibilidade em relação ao tempo disponível para a aplicação. No entanto, este trabalho apresenta a limitação de ser uma análise *in vitro*, o que impossibilita correlacionar a maior estabilidade da ED com um benefício clínico. Outro fator limitante é o número de variações técnicas existentes, como a angulação da válvula de três vias ou a inclusão de válvulas e filtros entre as seringas, o que dificulta a comparação entre técnicas. Sabe-se também que a concentração de POL, a proporção líquido + ar ambiente e a variação da temperatura da espuma em relação à temperatura corporal afetam negativamente a estabilidade da ED^25,26^ . Como perspectiva para futuros trabalhos, seria interessante comparar a influência da temperatura em concentrações inferiores a 1%.

## CONCLUSÃO

O resfriamento do conjunto POL a 1% + ar ambiente gerou aumento da meia-vida, independentemente da técnica de preparo utilizada. Não houve influência da temperatura no diâmetro das bolhas.
